# Management of secondary aorto-oesophageal fistula after aortic arch replacement: a case report without aortic reconstruction

**DOI:** 10.1093/ehjcr/ytag648

**Published:** 2026-08-31

**Authors:** Waka Saeki, Hirotsugu Kurobe, Tomohide Higaki, Taro Oshikiri, Hironori Izutani

**Affiliations:** Division of Surgery, Ehime University Hospital, 454 Shitsukawa, Ehime 791-0295, Japan; Department of Cardiovascular and Thoracic Surgery, Ehime University Graduate School of Medicine, 454 Shitsukawa, Ehime 791-0204, Japan; Department of Cardiovascular and Thoracic Surgery, Ehime University Graduate School of Medicine, 454 Shitsukawa, Ehime 791-0204, Japan; Department of Gastrointestinal Surgery and Surgical Oncology, Ehime University Graduate School of Medicine, 454 Shitsukawa, Ehime 791-0204, Japan; Department of Cardiovascular and Thoracic Surgery, Ehime University Graduate School of Medicine, 454 Shitsukawa, Ehime 791-0204, Japan

**Keywords:** Aorto-oesophageal fistula, Aortic arch replacement, Thoracic endovascular aortic repair, Oesophagectomy, Case report

## Abstract

**Background:**

Secondary aorto-oesophageal fistula (AEF) after aortic aneurysm repair is a rare but clinically catastrophic complication. Conservative management is associated with an unacceptably high risk of mortality, primarily due to severe infections. Although definitive *in situ* aortic reconstruction may be curative, it is highly invasive and carries substantial peri-operative morbidity and mortality, particularly in frail patients or those with established infection. This case report describes the successful treatment of a secondary AEF without aortic arch reconstruction, achieving durable long-term infection control.

**Case summary:**

A 74-year-old man was referred to our hospital with haematemesis, and a pseudoaneurysm was identified at the distal anastomosis site following total aortic arch replacement. Emergency thoracic endovascular aortic repair (TEVAR) was performed as a life-saving measure to achieve rapid haemostasis and stabilization. One month later, persistent fever and a residual oesophageal perforation prompted oesophagectomy with omental flap placement, without aortic arch reconstruction. Gastric conduit reconstruction was performed 4 months after TEVAR. The patient has remained event-free for more than 1 year after the initial presentation and intervention.

**Discussion:**

This case highlights the importance of an individualized treatment strategy for secondary AEF. In selected patients, infection control may be achieved without aortic arch reconstruction.

Learning points

- Secondary aorto-oesophageal fistula is a rare but life-threatening complication following aortic aneurysm repair that requires prompt diagnosis and management.
- In carefully selected frail or high-risk patients, staged TEVAR and oesophagectomy without aortic arch reconstruction may achieve durable infection control.
- Careful patient selection and long-term surveillance are essential for graft-preserving strategies.

## Introduction

Secondary aorto-oesophageal fistula (AEF) may occur when a pseudoaneurysm caused by anastomotic failure following aortic replacement compresses and perforates the oesophagus. Aorto-oesophageal fistula is a rare but life-threatening complication of aortic aneurysm surgical repair, with a reported incidence of 0.27%.^1^ Conservative management is associated with high mortality, largely because of severe infection, whereas *in situ* aortic reconstruction is highly invasive and carries increasing mortality risk.^1^

Thoracic endovascular aortic repair (TEVAR) can be life-saving by providing rapid haemorrhage control and haemodynamic stabilization in emergency situation, but it is not considered curative and requires additional intervention.^1^ Although several surgical strategies have been reported, including open aortic reconstruction or TEVAR combined with oesophagectomy and oesophageal reconstruction, the optimal management of secondary AEF remains undefined because of limited reported cases.^2^

This case describes the successful treatment of secondary AEF without aortic arch reconstruction, achieving durable long-term infection control.

## Summary figure

**Figure:**
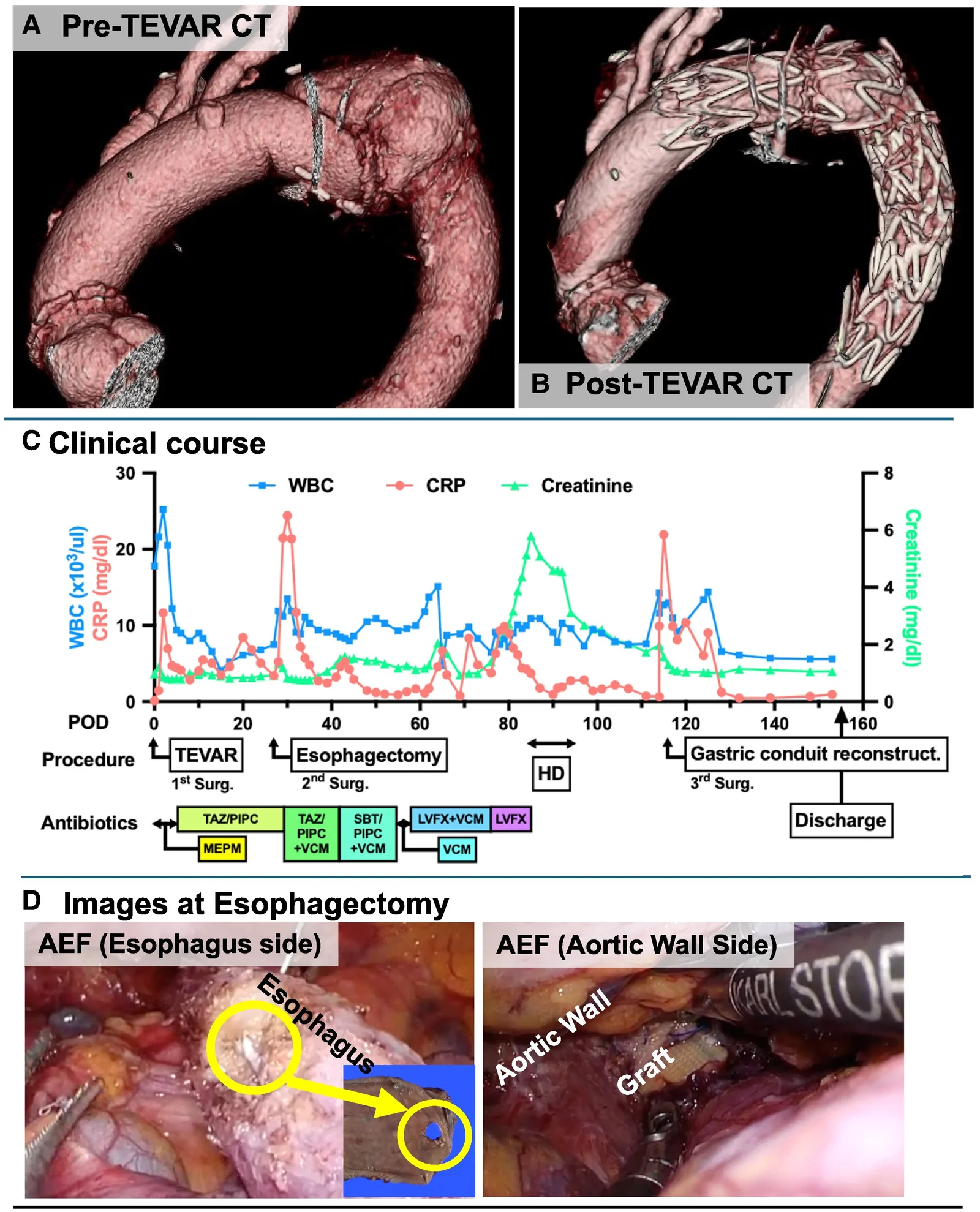


## Case presentation

A 74-year-old man was referred with haematemesis. At 54 years of age, he was diagnosed with Stanford type B acute aortic dissection and managed conservatively with antihypertensive therapy. At 67 years, he underwent aortic arch replacement and coronary artery bypass grafting at another hospital for an enlarging distal aortic arch aneurysm and pre-operatively diagnosed angina pectoris. He received no post-operative surveillance and continued antihypertensive therapy at a local clinic.

Genetic testing was not performed. Although syndromic aortopathy, including Marfan, Loeys–Dietz, and vascular Ehlers–Danlos syndromes, was considered, the patient had no clinical features suggestive of a heritable connective tissue disorder, and there was no family history of aortic disease.

**Figure 1:**
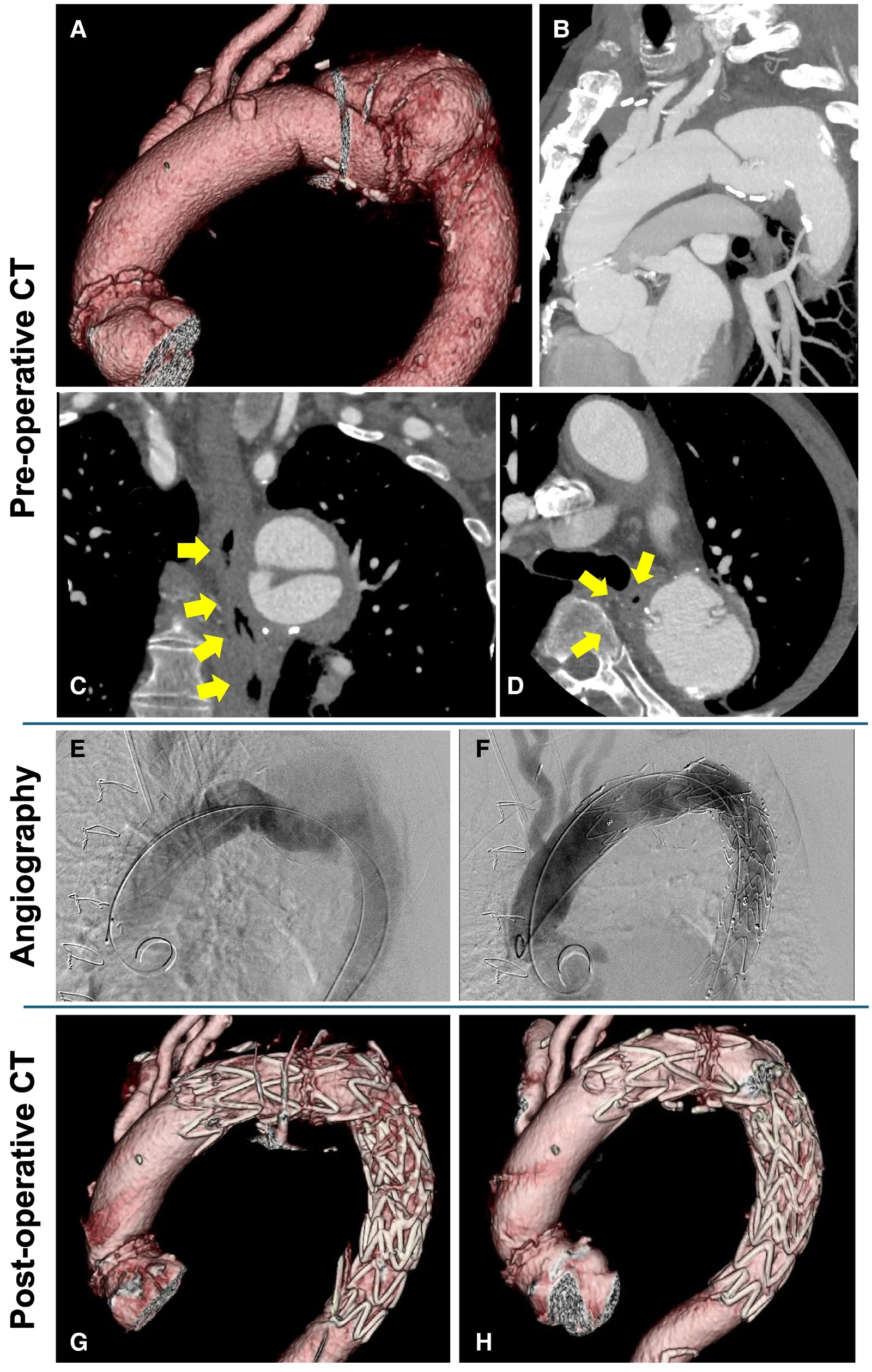
Computed tomography and aortography images. (*A–D*) Pre-operative computed tomography images showing a pseudoaneurysm measuring ∼7 cm at the distal anastomosis, with compression of the oesophagus (yellow arrows in *C* and *D*). (*E*) Peri-operative aortography demonstrating no extravasation outside the pseudoaneurysm. (*F*) Successful deployment of the stent grafts. (*G* and *H*) Post-operative CT following thoracic endovascular aortic repair confirming the absence of contrast leakage at 1 week (*G*) and 2 weeks (*H*). CT, computed tomography; TEVAR, thoracic endovascular aortic repair.

Approximately 10 years later, he developed chest pain and discomfort. Computed tomography (CT) revealed an enlarging pseudoaneurysm (∼7 cm) at the distal anastomosis compressing the oesophagus (*Figure 1A–D*). He subsequently developed massive haematemesis with haemodynamic shock.

He presented in a pre-shock state, with a blood pressure of 82/40 mmHg and a heart rate of 113 b.p.m. Laboratory findings were as follows: white blood cell count, 17 800/µL (reference: 3900–9800); red blood cell count, 260 × 10^4^/µL (reference: 427–570 × 10^4^); C-reactive protein, 0.13 mg/dL (reference: 0.00–0.20); and lactate, 28 mg/dL (reference: 5–12).

Emergency TEVAR covering the distal anastomosis was performed under general anaesthesia. The right femoral artery was exposed, and a purse-string suture was placed for device insertion. A 4 Fr Radifocus Introducer (Terumo Corporation, Japan) was inserted percutaneously into the left femoral artery for aortography. Two stent grafts (Valiant Thoracic; 28 mm × 100 mm, sized distally for the native descending aorta, and 36 mm × 150 mm, sized proximally for the artificial graft; Medtronic plc, Ireland) were deployed between the artificial graft and the descending aorta to exclude the pseudoaneurysm and achieve haemostasis and haemodynamic stabilization. Completion aortography demonstrated no extravasation outside the pseudoaneurysm (*Figure 1E* and *F*). Spinal drainage was not performed because of the emergency setting, and no post-operative neurological deficits were observed.

**Figure 2:**
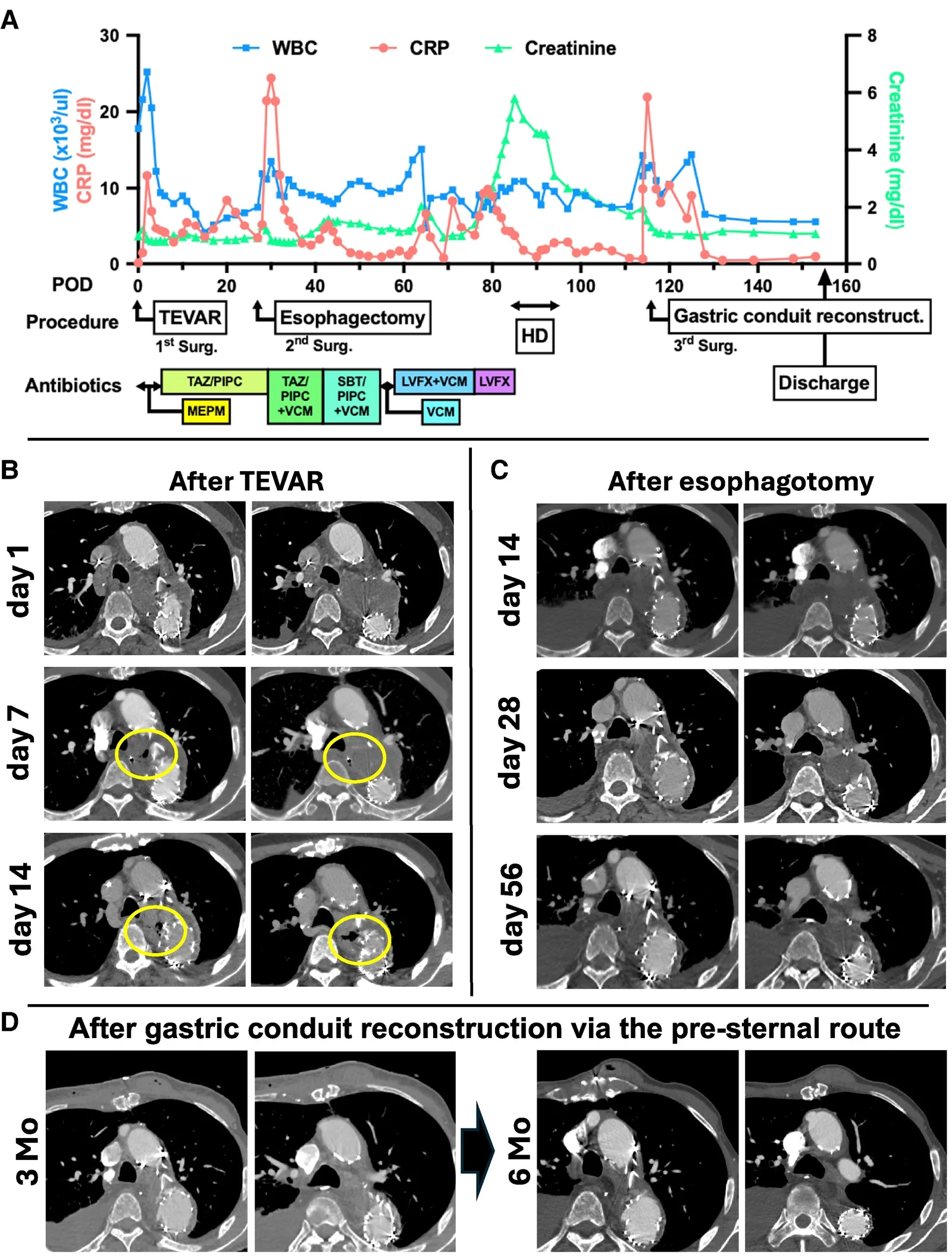
Computed tomography and aortography images. (*A*) The clinical course is shown. After thoracic endovascular aortic repair, antibiotic therapy was initiated. Despite the need for modification of antibiotics because of drug eruption and worsening renal function, inflammatory markers gradually improved after oesophagectomy. Antibiotics were discontinued 2 months after the second-stage surgery (∼3 months after thoracic endovascular aortic repair), and gastric conduit reconstruction via the pre-sternal route was performed at 4 months. The patient was discharged without evidence of recurrence. (*B*) A comparative computed tomography scan conducted after thoracic endovascular aortic repair demonstrates a progressive increase in the presence of free air within the mediastinum surrounding the oesophagus and pseudoaneurysm cavity (yellow circles). (*C*) After oesophagectomy, computed tomography confirms resolution of mediastinal free air. (*D*) Following gastric conduit reconstruction via the pre-sternal route, no recurrence is observable on computed tomography. HD, haemodialysis; CT, computed tomography; TEVAR, thoracic endovascular aortic repair; TAZ/PICC, tazobactam/piperacillin; MEPM, meropenem; VCM, vancomycin; SBT/PIPC, sulbactam/piperacillin; LVFX, levofloxacin.

The patient was managed under strict fasting, and empirical broad-spectrum intravenous meropenem (1 g every 12 h) was initiated in consultation with the infection control team according to the blood culture and clinical findings (*Figure 2A*). On post-operative Day 4, antibiotics were switched to tazobactam/piperacillin after blood cultures grew *Streptococcus constellatus* (*Figure 2A*).

**Figure 3:**
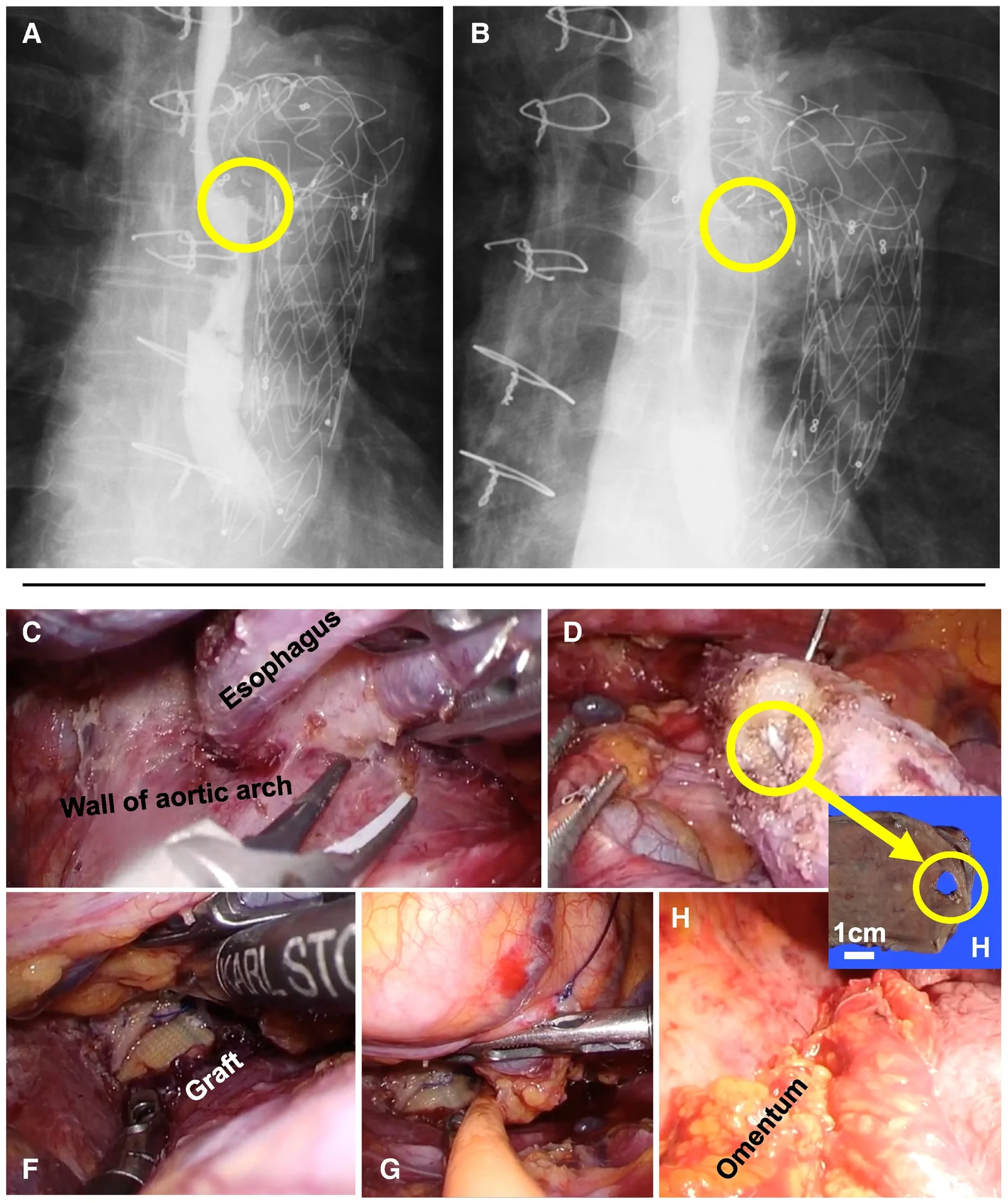
Computed tomography and aortography images. (*A* and *B*) Oesophagography demonstrating a persistent oesophageal perforation into the mediastinum (yellow circle). (*C*) Oesophagectomy was performed in a cranial-to-caudal direction, with careful dissection of the dense adhesions surrounding the aorto-oesophageal fistula. (*D*) The fistula between the oesophagus and pseudoaneurysm is exposed (yellow circle). (*E*) The diameter of the oesophageal perforation was ∼8 mm (yellow circle). (*F*) The artificial graft is shown. (*G*) After thorough debridement, the inflamed tissue was irrigated with saline. (*H*) The harvested omentum was placed around the artificial graft and within the mediastinum.

Although follow-up CT at 1 and 2 weeks demonstrated no contrast leakage (*Figure 1G* and *H*), free air volume within the pseudoaneurysm cavity and mediastinum progressively increased (*Figure 2B*). Oesophageal perforation-related infection was therefore suspected, and oesophagography confirmed a persistent AEF (*Figure 3A* and *B*). Definitive treatment options, including oesophagectomy with aortic arch reconstruction, were considered. However, considering the operative risk (EuroSCORE II-estimated operative mortality, 42.1%; Clinical Frailty Scale, 4), a staged surgical strategy was planned after discussion with the cardiac team. First, TEVAR was performed as life-saving procedure; second, oesophagectomy with omental placement into the pseudoaneurysm cavity and mediastinum were performed for infection control; third, delayed oesophageal reconstruction was performed after infection control. The patient and his family provided informed consent for this treatment plan.

Second-stage surgery was performed 4 weeks after TEVAR. Following omental harvest, a right posterolateral thoracotomy was performed through the fifth intercostal space. Oesophagectomy was performed in a cranial-to-caudal direction with careful dissection of the dense adhesions surrounding the AEF (*Figure 3C*). The AEF and artificial graft within the pseudoaneurysm were exposed (*Figure 3D–F*). Inflamed tissue surrounding the fistula was thoroughly debrided and irrigated with saline (*Figure 3G*), after which the harvested omentum was placed around the artificial graft to provide vascularized coverage and infection control (*Figure 3G*). Cervical oesophagostomy and enterostomy were then performed.

The patient was extubated on post-operative Day 1. Intra-operative cultures grew *Corynebacterium striatum.* Antibiotic therapy was modified because of drug eruption and deteriorating renal function; white blood cell count, C-reactive protein levels, and clinical findings improved (*Figure 2A*). Follow-up CT confirmed resolution of mediastinal free air (*Figure 2C*). Antibiotics were discontinued 2 months after the second-stage surgery following normalization of C-reactive protein levels and negative blood cultures.

Three months later, gastric conduit reconstruction was performed via the pre-sternal route. The patient was discharged 4 months after the second-stage surgery. He remained well for >1 year, with no evidence of recurrence on follow-up CT or blood tests (*Figure 2D*).

## Discussion

Three treatment strategies exist for secondary AEF: *in situ* aortic reconstruction with oesophagectomy, considered the gold standard;^1,3^ hybrid strategies combining TEVAR with oesophagectomy and oesophageal reconstruction; and conservative or palliative management for frail patients or those with high surgical risk.^4,5^ Each approach involves distinct trade-offs.

Newly aortic arch reconstruction combined with oesophagectomy offers the greatest likelihood of cure but requires cardiopulmonary bypass with deep hypothermic circulatory arrest and carries substantial peri-operative risk.^6^ In this patient, the combination of frailty (Clinical Frailty Scale 4), high predicted operative mortality (EuroSCORE II 42.1%), advanced age, and previous aortic arch replacement with coronary artery bypass grafting made this strategy unsuitable. Conversely, conservative management carries an unacceptably high risk of uncontrolled infection and recurrent haemorrhage.

Emergency TEVAR therefore provided rapid haemorrhage control and haemodynamic stabilization before definitive therapy. Aggressive multidisciplinary intervention with definitive oesophagectomy, aortic arch reconstruction, and oesophageal reconstruction can result in favourable long-term survival in selected patients without high risk.^7^ Similarly, patients with AEFs who underwent oesophagectomy had markedly longer survival than those who did not.^8^ In the present case, oesophagectomy with omental flap placement was performed 4 weeks after TEVAR to achieve definitive infection control.^3^ This staged strategy of TEVAR followed by oesophagectomy without aortic arch reconstruction balanced immediate haemostasis with source control while accepting retention of the prosthetic graft and the uncertain long-term risk of graft infection.

In the present case, durable infection control was achieved through debridement of inflamed tissue surrounding the AEF and omental coverage, without necessitating aortic arch reconstruction. Previously, in patients with infected thoracic aortic aneurysms, omental wrapping was associated with significantly improved 5-year infection-free survival.^5^ Omentum flap is believed to improve local infection control through its rich vascular supply, obliteration of dead space, and augmentation of local immune-mediated antimicrobial activuty.^9^ Beyond these mechanisms, preservation of infected prosthetic grafts combined with radical debridement and vascularized coverage is an established option in selected patients, with durable long-term preservation reported in most survivors and success strongly influenced by the virulence of the causative organism.^10^ In the present case, *C. striatum*, isolated from the intra-operative culture, was a relatively low virulence organism and may have facilitated infection control despite graft preservation.^11^

In the thoracic aorta, *in situ* preservation reinforced by omental wrapping has been associated with recurrence-free infection control beyond 2 years and, in some cases, up to a decade.^12^

Based on previous reports and the present case, a non-aortic reconstruction strategy may be considered only when the following criteria are met. First, the estimated operative mortality of aortic arch reconstruction is prohibitive based on objective risk assessment (e.g. EuroSCORE II together with the Clinical Frailty Scale). Second, the infective focus at the perforated oesophagus and surrounding inflamed tissue can be physically removed by oesophagectomy and radical debridement. Third, the causative organisms are of low virulence and antibiotic-susceptible. Finally, the prosthetic graft remains structurally intact on imaging, without radiological signs of active graft infection, such as peri-graft gas or abscess.

However, the long-term risk of re-infection remains uncertain. Therefore, long-term surveillance with CT, inflammatory marker, and blood cultures is essential to exclude late artificial graft infection. Recurrence of graft infection has been reported even after apparently successful graft preservation. Accordingly, the present strategy should be regarded as achieving durable infection control at 1 year rather than definitive cure.

Treatment decisions for AEFs are extremely challenging and require careful case-by-case assessment. This report adds to the limited evidence base for this rare and life-threatening condition rather than proposing a novel technique. Prospective multicentre registries are needed to establish evidence-based guidance for its management.

In conclusion, in frail, high-risk patients with a secondary AEF requiring emergency treatment, a staged strategy comprising emergency TEVAR, oesophagectomy with omentum wrapping, targeted antimicrobial therapy, nutritional optimization, and delayed oesophageal reconstruction may be a feasible treatment option in carefully selected patients. Quantitative operative risk and meticulous case-by-case evaluation are essential for patient selection, and structured long-term surveillance remains mandatory because the long-term risk of late graft infection is uncertain.

## Lead author biography


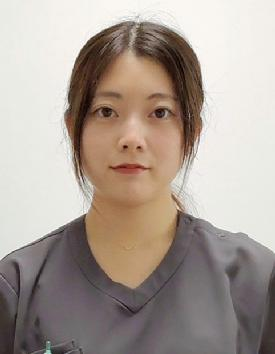
MD degree from Ehime University, Ehime, Japan, at 2025. Resident started at Ehime University Hospital in 2025.

## Data Availability

The data underlying this article will be shared on reasonable request to the corresponding author. No restricted data were used in this study.
